# Supplementary material for: A Likelihood Approach for Real-Time Calibration of Stochastic Compartmental Epidemic Models
Source: PLoS Comput Biol. 2017 Jan 17;13(1):e1005257. doi: 10.1371/journal.pcbi.1005257 (PMC5240920; doi:10.1371/journal.pcbi.1005257)
Supplement: S1 File — (TAR.GZ) [file pcbi.1005257.s014.tar.gz › HSPH_Online-SI-Revision/IPoi09/n100-extreme/n100-extreme_BENCH_summary.pdf]

peak -8	R0	$1/\theta_2$	inf	inflog	newly	newlylog	Reff	AttackRate
mean	23.9	44.3	127.6	0.5	229.1	0.6	24.	21.6
median	22.	42.	95.8	0.4	142.2	0.5	21.3	12.2
Quantile .1	10.3	15.1	60.3	0.4	63.5	0.4	10.	3.6
Quantile .9	41.7	77.9	222.4	0.8	505.	0.9	44.5	50.8
23	24	23						
length	50							

peak -4	R0	$1/\theta_2$	inf	inflog	newly	newlylog	Reff	AttackRate
mean	16.4	37.4	86.	0.3	72.7	0.3	22.5	7.4
median	10.5	22.	84.4	0.3	62.	0.3	19.2	3.9
Quantile .1	2.	4.	49.2	0.2	43.7	0.2	7.8	0.7
Quantile .9	35.7	76.3	127.3	0.4	110.3	0.5	40.2	18.2
7	5	7						
length	50							

peak	R0	$1/\theta_2$	inf	inflog	newly	newlylog	Reff	AttackRate
mean	24.4	39.6	40.5	0.2	21.2	0.1	46.	8.6
median	18.5	23.1	24.1	0.1	19.8	0.1	45.8	5.3
Quantile .1	3.8	2.6	14.9	0.1	14.1	0.1	38.9	0.8
Quantile .9	54.7	96.2	88.8	0.3	31.9	0.2	55.8	22.
0	0	1						
length	50							

peak +4	R0	$1/\theta_2$	inf	inflog	newly	newlylog	Reff	AttackRate
mean	23.7	42.1	141.3	0.3	75.1	0.2	36.2	8.4
median	20.	27.	56.8	0.2	48.4	0.2	38.2	5.7
Quantile .1	2.2	4.3	37.8	0.1	36.7	0.1	18.4	0.5
Quantile .9	48.	96.3	288.1	0.5	160.5	0.4	48.7	20.9
0	0	2						
length	50							

peak +8	R0	$1/\theta_2$	inf	inflog	newly	newlylog	Reff	AttackRate
mean	23.4	40.7	384.2	0.4	194.1	0.3	29.5	8.4
median	20.	27.	119.	0.3	91.4	0.3	28.2	5.6
Quantile .1	2.2	4.3	50.8	0.2	48.6	0.2	8.5	0.6
Quantile .9	54.7	96.2	604.1	0.8	328.1	0.6	54.5	21.
0	0	2						
length	50							
